# An Unusual Presentation of Cutaneous Bullous Lupus

**DOI:** 10.7759/cureus.74140

**Published:** 2024-11-21

**Authors:** Sara H Buchner, Kiana Malta, Shiyu Wang, Adriana Guevara, Cynthia Glickman, Kim Hookim, Pamela Traisak, Marissa Karpoff, David Feinstein, Hala Eid

**Affiliations:** 1 Orthopedics, Cooper Medical School of Rowan University, Camden, USA; 2 Psychiatry, University of Massachusetts Chan Medical School, Worcester, USA; 3 Rheumatology, Cooper University Health Care, Camden, USA; 4 Internal Medicine, Cooper University Health Care, Camden, USA; 5 Medicine, Cooper University Hospital, Camden, USA; 6 Pathology, Cooper University Health Care, Camden, USA; 7 Rheumatology, Cooper Medical School of Rowan University, Camden, USA; 8 Rheumatology, Cooper University Hospital, Cooper Medical School, Camden, USA

**Keywords:** bullous lesion, bullous systemic lupus erythematosus (bsle), drug-induced lupus (dil), hydralazine, systemic lupus erythematosus

## Abstract

Drug-induced lupus erythematosus (DILE) is an autoimmune reaction that results in symptoms of polyarthralgia, fever, and cutaneous lesions and other manifestations. Several drugs have been documented to cause this disease, including procainamide, isoniazid, methyldopa, penicillamine, and hydralazine. Systemic lupus erythematosus (SLE) manifestations often occur after the patient has been taking the drug without complications for months to years. Hydralazine, an oral antihypertensive drug, is a well-known cause of DILE; however, clinical presentation is generally limited to classic SLE symptoms such as malar or discoid rash and polyarthralgia. In this case, we will discuss a 75-year-old female patient with actively bleeding oral, nasal, and conjunctival ulcers of unknown origin, eventually found to have bullous lupus erythematosus while on hydralazine for several years. Bullous lupus erythematosus is a rare cutaneous manifestation of SLE that presents with blister-type lesions in patients with a history of SLE. Histologically, bullous lupus erythematosus is characterized by marked epidermal and dermal inflammation, and an inflammatory infiltrate that consists primarily of neutrophils at the base of the bullae. Although hydralazine has been implicated in DILE, it is very rarely noted to have a bullous lupus erythematosus presentation. Given the acuity of patient’s presentation and resolution with cessation of hydralazine, it was concluded that hydralazine was the main contributing factor for this patient’s multiple hemorrhagic bullous lupus erythematosus lesions.

## Introduction

Systemic lupus erythematosus (SLE) is an autoimmune disease in which immune complexes attack healthy cells throughout the body, resulting in various systemic signs, generally manifesting as constitutional, cutaneous, articular, neurologic, serositis, hematologic, and renal symptoms [1]. Although most cases of SLE are idiopathic, over 100 drugs have been documented to induce SLE [2,3], with an estimated 15,000-30,000 new cases of drug-induced lupus erythematosus (DILE) occurring every year in the United States [4]. DILE occurs after months to years of exposure to certain drugs and generally resolves after the cessation of the medication [3].

Medications have been grouped into classes based on the degree of risk they pose for causing DILE. Drugs with a low risk of causing DILE include minocycline and tumor necrosis factor alpha (TNF-alpha) inhibitors, whereas isoniazid poses moderate risk for DILE. Procainamide and hydralazine are considered high-risk DILE medications [3]. Hydralazine, a potent vasodilator used to treat hypertension, poses an 8-13% risk of developing DILE [4]. All these drugs can lead to the development of SLE symptoms or cause lupus flares in pre-existing SLE [5].

Even less common than drug-induced SLE is drug-induced bullous lupus erythematosus (DIBL). Bullous lupus is a rare manifestation of SLE in which patients present with subepidermal blisters that appear in small clusters or as large, tense bullae [6]. These lesions can appear anywhere but most commonly appear on the face, neck, upper torso, supraclavicular area, axillae, proximal extremities, and mucous membranes [7]. This blistering is caused by the formation of autoantibodies against type VII collagen, which weakens the connection between the dermis and epidermis resulting in the formation of bullae [7]. Bullous lupus most commonly affects women, with the highest incidence in women of African descent [7]. Because of its rarity, the prevalence of bullous lupus is difficult to estimate, but large cohort studies hypothesize that 0.19-0.41% of patients with SLE develop a bullous lupus manifestation [7]. Case reports associate TNF-alpha inhibitors, interleukin-6 (IL-6) inhibitors, terbinafine, methimazole, and penicillamine with DIBL [2,6,8,9].

Cutaneous manifestations are present in one-third of patients with hydralazine DILE; however, this is usually restricted to the classic malar or discoid rash seen in SLE [4]. There have been very few reports of a patient who had been taking hydralazine presenting with bullous lupus-like lesions over the course of several decades [2,10,11].

## Case presentation

**Figure 1:**
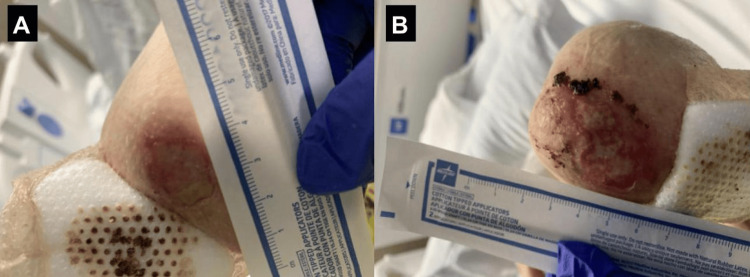
Erupted 2 cm (A) and 4 cm (B) bullous lesions on the upper extremity

**Figure 2:**
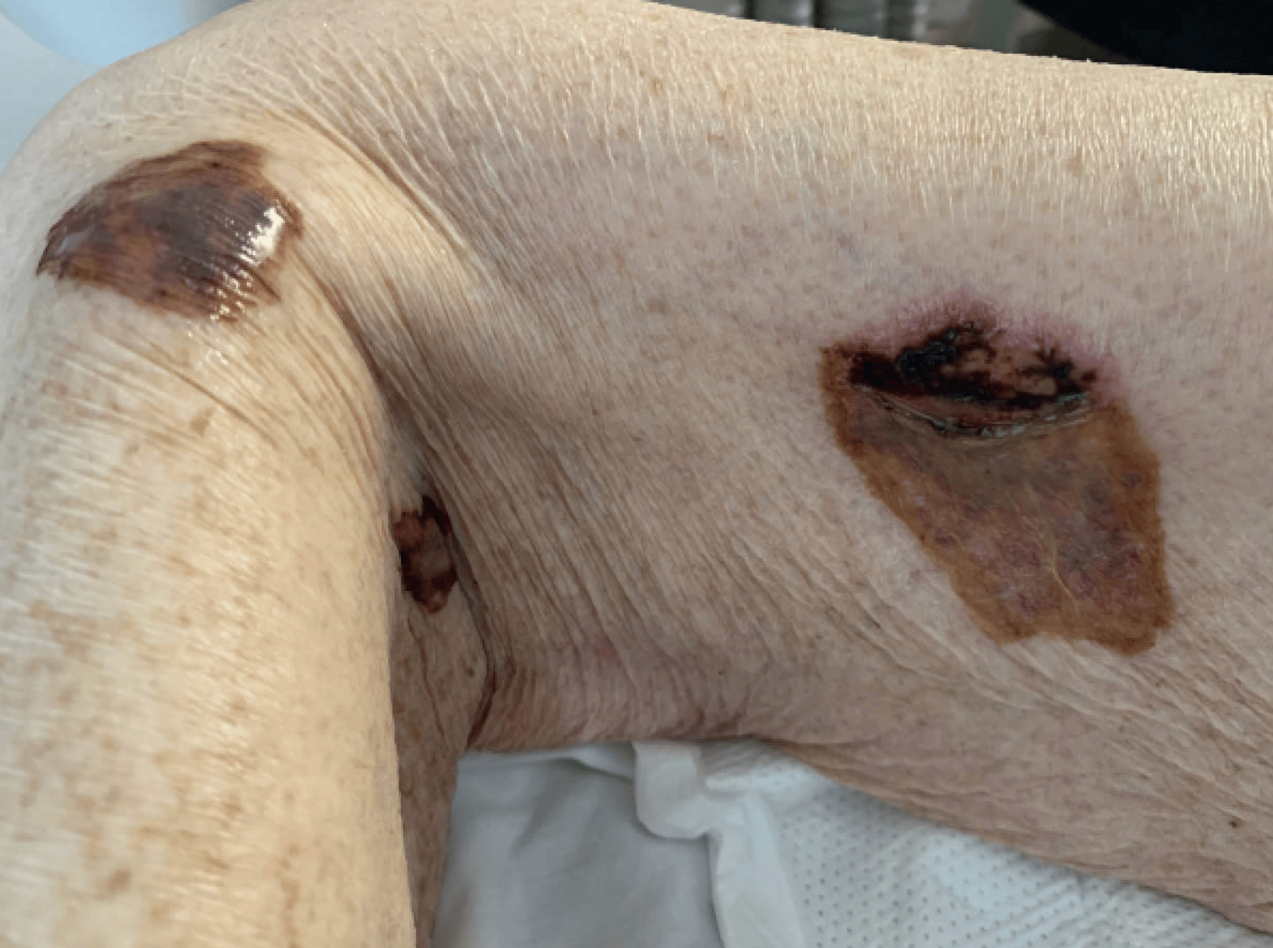
Lower extremity bullous lesions

**Figure 3:**
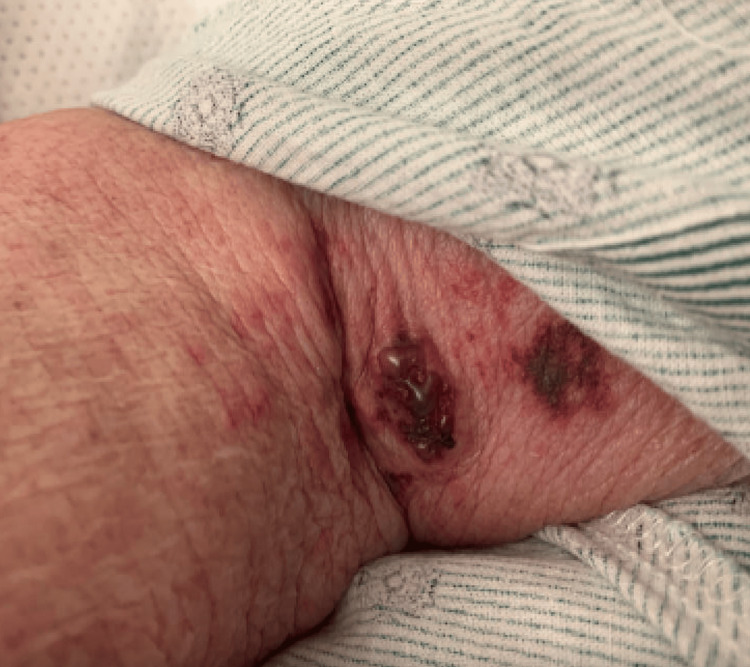
Intact hemorrhagic bullous lesion

A 75-year-old female patient with past medical history of group 5 pulmonary hypertension (pHTN), atrial fibrillation (Afib), hypertension (HTN), hyperlipidemia, hypothyroidism, coronary artery disease, and SLE diagnosed about one year prior to hospital admission presented with oral, nasal, and conjunctival hemorrhage, along with widespread polyarthralgia and varying skin wounds and lesions diffusely on the upper and lower extremities bilaterally (Figures 1-3). The patient had difficulties with speech, breathing, and oral intake due to hemorrhage and skin breakdown upon admission. Articular manifestations included tenderness and swelling of the bilateral elbow, shoulder, wrist, metacarpophalangeal, and ankle joints. Home medications included apixaban 5 mg twice daily for Afib and hydralazine 25 mg twice daily for HTN, which the patient had been taking for over 10 years.

Our patient was diagnosed with SLE by an outside rheumatologist about one year prior to this hospitalization. She met the criteria for SLE diagnosis based on the ACR 2019 criteria of positive antinuclear antibodies (ANA), low complements (C3 and C4), and elevated double stranded DNA (dsDNA) along with articular manifestations. Her prior lupus flares involved skin rashes, joint pains, and occasional mucosal ulcerations but were never associated with bleeding or cutaneous symptoms. Initial dermatology evaluation described the lesions as purpuric nodules with central ulceration on the eyelid margins, nose, lips, and mucosal surface of the mouth. There was also retiform purpura, hemorrhagic bullae on the bilateral forearms, and inflammatory purpura on the left lower extremity.

Pertinent labs included: positive ANA (1:1280), elevated dsDNA antibodies at 12 (negative </= 4 dsDNA), decreased C3 (68 mg/dL) and C4 (16 mg/dL) levels and elevated anti-histone antibodies at 3.4 units (negative <1 unit). In addition, myeloperoxidase (MPO) antibodies were elevated (155.1 U/mL), as well as anti-neutrophil cytoplasmic (p-ANCA) antibody levels (1:1640) (Table 1). The patient was also found to have prolonged PT (19.1 s), PTT (41.5 s), INR (1.7 s), factor IX (245 U/dL), and factor X (57 U/dL), with these results suggesting a potential factor inhibitor, which is not unusual for patients over 70 years with SLE. In the setting of prolonged PT and PTT, antiphospholipid antibody work-up was initiated, which revealed negative cardiolipin IgA/IgM/IgG, negative beta 2 glycoprotein IgA/IgM/IgG, and positive lupus anticoagulant hexagonal phase phospholipid neutralization.

**Table 1:** Patient lab values

	Patient labs	Reference range
Antinuclear antibodies (ANA)	1:1280	= 1:40
Anti-neutrophil cytoplasmic (p-ANCA) antibodies	1:1640	< 1:20
Anti-double-stranded DNA (dsDNA) antibodies	12 units	= 4 units
Anti-histone antibodies	3.4 units	< 1 unit
Anti-myeloperoxidase (MPO) antibodies	155.1 U/mL	< 1 U/mL
C3	68 mg/dL	100-233 mg/dL
C4	16 mg/dL	14-48 mg/dL
Prothrombin time (PT)	19.1 seconds	11-13 seconds
Activated partial thromboplastin time (aPTT)	41.5 seconds	25-35 seconds
INR	1.7	0.8-1.1
Factor IX	245 U/dL	50-150 U/dL
Factor X	57 U/dL	50-200 U/dL

The biopsy from the left arm showed marked dermal and epidermal acute inflammation with intracorneal neutrophils, focal epidermal necrosis and subepidermal separation, compatible with bullous lupus erythematosus. Direct immunofluorescence examination of the left arm revealed prominent staining of the superficial dermal blood vessels for IgM and C3, consistent with necrotizing vasculitis. Histological features of vasculitis were not seen in the left arm biopsy but were confirmed in an additional biopsy taken from the left thigh which showed features of acute necrotizing vasculitis.

Due to concerns for a severe lupus flare, IV methylprednisolone 60 mg daily was started. Hydralazine was also discontinued as it was considered the likely cause of the SLE flare and bullous lupus erythematous lesions. Over the course of her admission, the patient began to show significant improvements in methylprednisolone. Her skin lesions began to improve as did her ability to ambulate and communicate. When she was able to tolerate oral medication, intravenous methylprednisolone was discontinued, and she was switched to oral prednisone 50 mg daily and mycophenolate mofetil 500 mg twice daily. The patient was discharged on oral prednisone taper and mycophenolate mofetil 500 mg twice daily. Three months later on outpatient follow-up, the patient’s skin lesions had nearly fully resolved without recurrence, and there was only minor arthralgias without other new symptoms. Lab values over a year and a half after admission have remained within normal limits, including anti-dsDNA (1 unit), C3 levels (110 mg/dL), and C4 levels (28 mg/dL). Her p-ANCA antibodies also normalized (<1:20), and her anti-MPO antibodies have remained only slightly elevated (6.4 units).

## Discussion

Hydralazine is a high-risk drug for inducing DILE [3], but cutaneous manifestations are rare [4]. The exact prevalence of bullous manifestations of SLE has been difficult to determine due to its rarity, making our patient’s presentation of hydralazine-induced bullous lupus notable.

Although idiopathic SLE and DILE present very similarly, there are several distinguishing features. Importantly, DILE is more common in the elderly than SLE. Central nervous system involvement and malar rash are commonly seen in SLE but rarely seen in DILE. In addition, anemia, leukopenia, and thrombocytopenia are less common in DILE [4]. Patients with DILE have ANA in 90-100% of cases and anti-histone antibodies in 90-95% of cases [5] but do not usually have the hypocomplementemia or anti-dsDNA antibodies that are characteristic of SLE. In rare cases, patients with DILE also develop ANCA, specifically p-ANCA or MPO antibodies, as seen in our patient [4].

The exact mechanism by which hydralazine induces SLE is not fully understood, but the majority of patients with hydralazine-induced lupus are slow acetylators, making them prone to the accumulation of autoantibodies [3]. Studies show that hydralazine alters the calcium levels within neutrophils, which triggers the formation of neutrophil extracellular traps, a major source of autoantigens [4].

The criteria for diagnosis of bullous SLE are as follows: (1) features or diagnosis of SLE based on the American College of Rheumatology (ACR) 2019 criteria; (2) Acutely acquired vesiculobullous rash; (3) Histopathological evidence of subepidermal blisters and dermal infiltrate consisting mostly of neutrophils; (4) Direct immunofluorescence demonstrating deposition of IgG, IgM, or IgA at the basement membrane zone (BMZ); (5) Evidence of antibodies to type VII collagen; (6) Exclusion of other blistering disorders [7]. In addition to blisters and anti-type VII collagen antibodies (anti-NC1 and anti-NC2), patients with bullous lupus have similar lab findings to those with SLE, including ANA, anti-dsDNA antibodies, anti-Smith antibodies, anemia, leukopenia, thrombocytopenia, and hypocomplementemia [7]. Diagnosis also requires confirmatory histopathology, which shows separation of the dermis from the epidermis at the BMZ and a primarily neutrophilic inflammatory infiltrate in the upper dermis. In addition, direct immunofluorescence staining (DIF) shows linear labeling with anti-IgG. Similar deposition with anti-IgM, IgA, and C3 may also be seen [7]. Pathology consistent with necrotizing vasculitis is also not uncommon [7].

Our patient with a one-year history of SLE presented to our facility with an acutely acquired vesiculobullous rash, with purpuric nodules and central ulceration on the eyelid margins, nose, lips, and mucosal surface of the mouth, as well as retiform purpura with overlying hemorrhagic bullae on the bilateral forearms. She also had inflammatory purpura on the left lower extremity, all of which is consistent with bullous SLE. The histological manifestations seen in our patient also aligned with the diagnostic requirements for bullous SLE. Biopsy of her hemorrhagic bullae showed marked epidermal and dermal acute inflammation with intracorneal neutrophils, focal epidermal necrosis, and subepidermal separation. The biopsy was negative for acantholysis or vasculitis. DIF results showed a vascular reaction pattern, consistent with necrotizing vasculitis.

Other autoimmune blistering conditions were excluded by the absence of immunofluorescent reactivity at the dermo-epidermal junction, and supportive serological findings. For example, although bullous skin lesions may present similarly in bullous pemphigoid, DIF would show antibody deposition on the epidermal side of skin splitting, which is not seen in our patient. Similarly, Stevens-Johnson syndrome (SJS), a severe cutaneous adverse drug reaction, can be differentiated from bullous SLE via histologic findings. SJS presents with widespread epidermal necrosis involving all skin layers, which is not seen here [12]. In addition, this patient’s symptoms of joint pain and swelling and confirmed diagnosis of SLE make other blistering disorders, such as SJS and infectious etiology, such as bullous impetigo, less likely.

The trigger for this patient’s bullous SLE flare was determined to be from hydralazine. Our patient’s antibody profile was positive for anti-histone antibodies, along with positive p-ANCA and MPO antibodies. Patients with DIL can develop p-ANCA or MPO antibodies in some cases [4], which corresponds to this patient’s elevation in MPO antibodies (155.1). Anti-histone antibodies are also present in up to 75% of patients with DILE, with a sensitivity of 55-92% and a specificity of 69-82%. Our patient had been taking hydralazine 25 mg for several years before this lupus flare, which she noted was different from prior lupus flares in that she had never experienced any plaques or mucosal bleeding. DIL symptoms do not usually appear before three years of taking the medication [4]. There have been other reports of late-onset DILE [13], but because our patient was diagnosed with SLE by an outside rheumatologist one year prior to hospitalization, we cannot say for sure whether this is a case of late-onset DILE. Lastly, our patient experienced a gradual resolution in symptoms upon withdrawal of hydralazine, which is a major distinguishing factor between DIL and idiopathic SLE [4]. Although administration of methylprednisolone and mycophenolate mofetil may have contributed to her improvement in the acute phase, she continues to have resolution of skin symptoms with discontinuation of these medications, which is consistent with a drug-induced reaction.

## Conclusions

Limitations of this study include lack of data on the patient’s anti-basement membrane zone antibody, anti-intercellular substance antibody, anti-NC1, and anti-NC2 status. These tests were not readily available at our facility and the patient was in urgent status initially. In addition, DIF imaging was unable to be provided for this patient because images were not saved; only a report of pathology was provided. This case highlights the potential of hydralazine to induce severe and rare bullous manifestations in patients with a history of SLE. Given that this patient’s bullous lupus flare compromised her ability to breathe and eat, it should be considered that hydralazine poses a potential risk of producing life-threatening complications in patients with a history of SLE.
